# Fees

**Published:** 1904-03

**Authors:** W. C. Trotter


### FEES.

It is an impossibility to set down any arbitrary scale of fees. I think that a dentist should receive at least four dollars per working hour for time and material. Necessary treatments should be charged for at the rate of fifty cents each. For ordinary cleaning with pumice, etc., one dollar is a fair charge. Where deposits are removed with scalers, I think a minimum charge of two dollars should be made, but such operations as this should be charged for according to the time consumed, at not less than four dollars per hour. An adequate fee for crown work of any description is ten dollars per tooth, and should not be less than eight dollars. I do not believe any man can afford to pay proper attention to the details of making a set of artificial teeth for less than ten dollars or of resetting them for less than five dollars. A repair, unless very simple, should be worth two dollars. Gold fillings should be charged for according to the time consumed. Alloy fillings at one dollar each are reasonable enough. Devitalizing and filling roots, together with an alloy filling should certainly not be done for less than three dollars.—*Dr. W. C. Trotter, Dom. Journal.*

